# A nationwide randomized controlled trial on additional treatment for isolated local pancreatic cancer recurrence using stereotactic body radiation therapy (ARCADE)

**DOI:** 10.1186/s13063-022-06829-1

**Published:** 2022-10-28

**Authors:** I. W. J. M. van Goor, L. A. Daamen, M. G. Besselink, A. M. E. Bruynzeel, O. R. Busch, G. A. Cirkel, B. Groot Koerkamp, N. Haj Mohammed, H. D. Heerkens, H. W. M. van Laarhoven, G. J. Meijer, J. Nuyttens, H. C. van Santvoort, G. van Tienhoven, H. M. Verkooijen, J. W. Wilmink, I. Q. Molenaar, M. P. W. Intven, for the Dutch Pancreatic Cancer Group

**Affiliations:** 1 Department of Surgery, Regional Academic Cancer Center Utrecht, Utrecht, the Netherlands; 2 Nieuwegein, the Netherlands; 3 Department of Radiation Oncology, Regional Academic Cancer Center Utrecht, Utrecht, the Netherlands; 4 Division of Imaging and Oncology, University Medical Center Utrecht, Utrecht, the Netherlands; 5 Department of Surgery, Amsterdam University Medical Center, University of Amsterdam, Amsterdam, the Netherlands; 6 Cancer Center Amsterdam, Amsterdam, the Netherlands; 7 Department of Radiation Oncology, Amsterdam University Medical Center, location Vrije Universiteit, Amsterdam, the Netherlands; 8 Department of Medical Oncology, Regional Academic Cancer Center Utrecht, Utrecht, the Netherlands; 9 Department of Surgery, Erasmus Medical Center, Rotterdam, the Netherlands; 10 Utrecht, the Netherlands; 11 Department of Radiation Oncology, Radboud University Medical Center, Nijmegen, the Netherlands; 12 Department of Medical Oncology, Amsterdam University Medical Center, location University of Amsterdam, Amsterdam, the Netherlands; 13 Department of Radiation Oncology, Erasmus Medical Center, Rotterdam, the Netherlands; 14 Department of Radiation Oncology, Amsterdam University Medical Center, University of Amsterdam, Amsterdam, the Netherlands

**Keywords:** Pancreatic cancer, Pancreatic ductal adenocarcinoma, PDAC, Disease recurrence, Isolated local recurrence, Stereotactic body radiation therapy, SBRT, SABR, Image-guided radiotherapy

## Abstract

**Background:**

Disease recurrence is the main cause of mortality after resection of pancreatic ductal adenocarcinoma (PDAC). In 20–30% of resected patients, isolated local PDAC recurrence occurs. Retrospective studies have suggested that stereotactic body radiation therapy (SBRT) might lead to improved local control in these patients, potentially having a beneficial effect on both survival and quality of life. The “nationwide randomized controlled trial on additional treatment for isolated local pancreatic cancer recurrence using stereotactic body radiation therapy” (ARCADE) will investigate the value of SBRT in addition to standard of care in patients with isolated local PDAC recurrence compared to standard of care alone, regarding both survival and quality of life outcomes.

**Methods:**

The ARCADE trial is nested within a prospective cohort (Dutch Pancreatic Cancer Project; PACAP) according to the ‘Trials within Cohorts’ design. All PACAP participants with isolated local PDAC recurrence after primary resection who provided informed consent for being randomized in future studies are eligible. Patients will be randomized for local therapy (5 fractions of 8 Gy SBRT) in addition to standard of care or standard of care alone. In total, 174 patients will be included. The main study endpoint is survival after recurrence. The most important secondary endpoint is quality of life.

**Discussion:**

It is hypothesized that additional SBRT, compared to standard of care alone, improves survival and quality of life in patients with isolated local recurrence after PDAC resection.

**Trial registration:**

ClinicalTrials.gov registration NCT04881487. Registered on May 11, 2021.

## Administrative information

Note: the numbers in curly brackets in this protocol refer to SPIRIT checklist item numbers. The order of the items has been modified to group similar items (see http://www.equator-network.org/reporting-guidelines/spirit-2013-statement-defining-standard-protocol-items-for-clinical-trials/).

**Table:** 

Title {1}	A Nationwide Randomized Controlled Trial on Additional Treatment for Isolated Local Pancreatic Cancer Recurrence using Stereotactic Body Radiation Therapy (ARCADE)
Trial registration {2a and 2b}.	Clinicaltrials.gov registration, NCT04881487. Registered on May 11, 2021, https://www.clinicaltrials.gov/ct2/show/NCT04881487.
Protocol version {3}	Version 2.0, February 4, 2021.
Funding {4}	Dutch Cancer Society (KWF; grant number 12568).
Author details {5a}	^1^ Department of Surgery, Regional Academic Cancer Center Utrecht, Utrecht, the Netherlands. ^2^ Nieuwegein, the Netherlands. ^3^ Department of Radiation Oncology, Regional Academic Cancer Center Utrecht, Utrecht, the Netherlands. ^4^ Division of Imaging and Oncology, University Medical Center Utrecht, Utrecht, the Netherlands. ^5^ Department of Surgery, Amsterdam University Medical Center, University of Amsterdam, Amsterdam, the Netherlands. ^6^ Cancer Center Amsterdam, Amsterdam, the Netherlands. ^7^ Department of Radiation Oncology, Amsterdam University Medical Center, location Vrije Universiteit, Amsterdam, the Netherlands. ^8^ Department of Medical Oncology, Regional Academic Cancer Center Utrecht, Utrecht, the Netherlands. ^9^ Department of Surgery, Erasmus Medical Center, Rotterdam, the Netherlands. ^10^ Utrecht, the Netherlands. ^11^ Department of Radiation Oncology, Radboud University Medical Center, Nijmegen, the Netherlands. ^12^ Department of Medical Oncology, Amsterdam University Medical Center, location University of Amsterdam, Amsterdam, the Netherlands. ^13^ Department of Radiation Oncology, Erasmus Medical Center, Rotterdam, the Netherlands. ^14^ Department of Radiation Oncology, Amsterdam University Medical Center, University of Amsterdam, Amsterdam, the Netherlands.
Name and contact information for the trial sponsor {5b}	University Medical Center Utrecht Heidelberglaan 100 PO Box 85500 3584 CX Utrecht, the Netherlands
Role of sponsor {5c}	The sponsor is responsible for setting up the trial, trial coordination, data collection and analysis and submitting the final manuscript for publication.

## Introduction

### Background and rationale {6a}

#### Background

Pancreatic ductal adenocarcinoma (PDAC) currently is the fourth leading cause of cancer-related death for both men and women [1–3]. For patients with localized, resectable disease, surgery combined with (neo)adjuvant therapy offers the best chance for long-term survival [4–6]. However, even after resection, almost all patients develop local and/or distant disease recurrence, mostly within the first 2 years [6–10]. Therefore, PDAC continues to be associated with a 5-year survival of only 12–17% after resection [11–14].

Local recurrence without evidence of distant metastases (also known as isolated local recurrence) occurs in 20–30% of all patients with PDAC recurrence [15–20]. These patients have a slightly better prognosis with a median time to recurrence of 9 months, compared to 7 months in case of distant metastases. Also, better median survival after recurrence is reported, with respectively 9 compared to 6 months [4, 10, 21].

Currently, most patients with PDAC recurrence and a sufficient performance status are treated with palliative chemotherapy as survival is predominantly determined by systemic disease control [22]. Isolated local PDAC recurrence, however, is frequently associated with considerable morbidity from local destructive tumor growth, including pain, gastrointestinal or biliary obstruction, malnutrition and portal hypertension, resulting in a significantly decreased quality of life [23]. Local therapy therefore might be of additional value to improve local disease control, which could positively improve quality of life in these patients [21, 24–30]. Additionally, previous studies suggest survival benefit of additional local therapy [22].

Radiation therapy is a widely accepted treatment modality for various types of cancers, being a minimally invasive therapeutic option with a relatively mild toxicity profile [31, 32]. The main difficulty with radiation therapy in PDAC patients, however, is that the pancreas is tightly surrounded by organs with limited radiation dose tolerance, such as the duodenum, small bowel, and stomach [33]. Besides, day to day position variation and motion of the structures in the upper abdomen due to respiration and bowel filling increases the required margins, hence enhancing the need for dose restriction [34]. These factors impede high-dose irradiation of tumors in the pancreas and peri-pancreatic region [35]. However, it is suggested that high-dose irradiation is required to achieve local control of the PDAC recurrence [36, 37].

In recent years, novel radiotherapy techniques, such as image-guided stereotactic body radiation therapy (SBRT), have been introduced that allow delivery of high-dose irradiation to the pancreatic tumor while limiting exposure of normal adjacent organs and tissues [38–42]. SBRT is a method of external beam radiation therapy that accurately delivers a high irradiation dose to a target in a limited number of fractions under guidance of online CT or MR imaging. It compensates the motion and position variation of the target by following it real time (tracking) or having the beam on only if the tumor is in the right spot (gating). It was demonstrated that SBRT safely enables dose escalated radiotherapy to pancreatic lesions. In the Netherlands, the University Medical Center (UMC) Utrecht with the MR-linac, the Amsterdam UMC with the MRIdian and the Erasmus Medical Center (MC) with the Cyberknife all have extensive experience with delivering SBRT to the pancreatic region [43–45]. Early results of retrospective single-center studies suggest improved progression-free survival, which could be translated to longer overall survival [37, 42, 46]. Moreover, patients with isolated local PDAC recurrence showed a median survival after SBRT of up to 16 months [22, 47, 48]. Additionally, SBRT improved local control and palliation of symptoms related to local progression in PDAC patients [47, 49, 50]. In patients with local PDAC recurrence treated with SBRT, treatment-related toxicity was relatively mild with 10% experiencing a grade 3 or higher gastrointestinal toxicity [47]. However, prospective studies are needed to confirm the value of SBRT in patients with isolated local PDAC recurrence, in addition to standard of care.

#### Rationale

The ARCADE trial aims to investigate whether additional local treatment using SBRT improves survival and quality of life in patients with isolated local recurrent PDAC, compared to standard of care alone.

### Objectives {7}

The hypothesis of the ARCADE trial is that local SBRT, in addition to standard of care, may improve local control, survival, and quality of life, with acceptable toxicity, in patients with an isolated local recurrence of pancreatic cancer. The main objective of this study is to improve survival after recurrence in these patients.

The secondary objectives of this study are:

- To assess impact of SBRT on quality of life
- To assess impact of SBRT on overall survival
- To assess impact of SBRT on disease-free survival
- To assess impact of SBRT on local progression-free interval
- To assess impact of SBRT on distant metastasis-free interval
- To assess treatment response by computed tomography (CT) imaging
- To assess acute and late toxicity of SBRT
- To assess patients’ acceptability of SBRT

### Trial design {8}

The ARCADE trial is a nationwide, multicenter trial embedded in the large nationwide prospective cohort (the Dutch Pancreatic Cancer Project; PACAP). PACAP serves as a trial facility following the “Trials within Cohorts” (TwiCs) design [51, 52]. With this design, “a large observational cohort of patients is recruited and used as a multiple trials facility and ‘patient centered’ information and consent are applied” [53, 54].

**Fig. 1:**
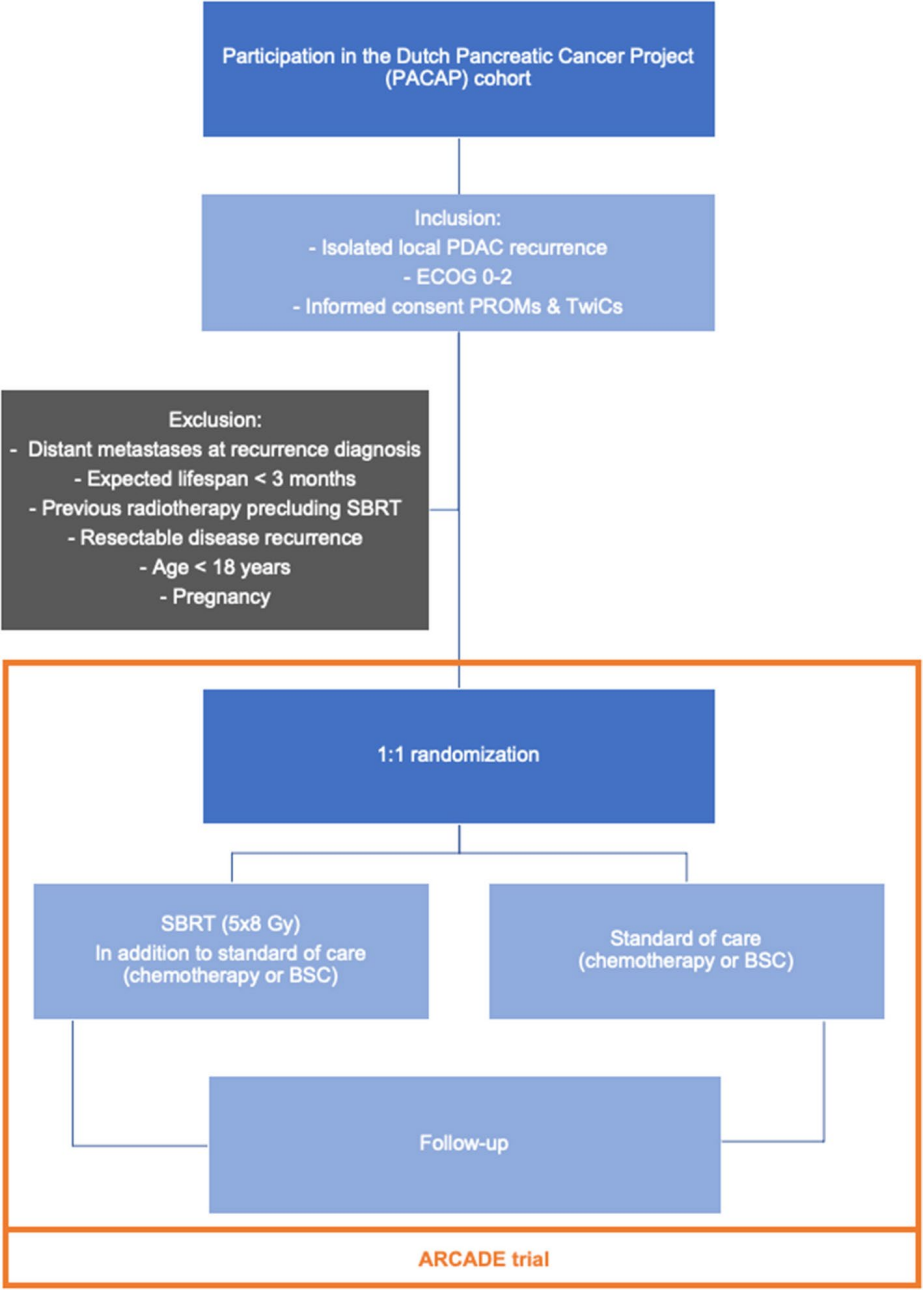
Flowchart of the ARCADE study process. PDAC, pancreatic ductal adenocarcinoma; ECOG, Eastern Cooperative Oncology Group; PROMs, patient-reported outcome measures; TwiCs, Trials within Cohorts; MRI, magnetic resonance imaging; CT, computed tomography; SBRT, stereotactic body radiation therapy; Gy, gray; BSC, best supportive care

The local investigator or an authorized delegate will check whether the patient meets all inclusion criteria (and none of the exclusion criteria). The patients who are eligible to participate and provided broad informed consent for participation in the PACAP cohort, quality of life questionnaires, and future randomization will form a subcohort. From this subcohort, patients are randomized by a computer-generated randomization module integrated in the Castor Electronic Data Capture (EDC) system in a 1:1 ratio for either the intervention or the control group. Patients randomly selected for the intervention are consulted by the radiation oncologist from one of the three participating radiation centers who will give them information about the intervention. Afterwards, patients decide whether they want to undergo the intervention and if they decide to do so, additional written informed consent is obtained (staged informed consent) [55]. Patients allocated to the control arm are not informed and undergo standard treatment, and their outcomes, collected in the context of PACAP, will be used comparatively. To illustrate, the inclusion and randomization process of the ARCADE trial is schematically shown in Fig. 1.

## Methods: participants, interventions, and outcomes

### Study setting {9}

All patients with isolated local PDAC recurrence, who participate in the PACAP cohort and have provided informed consent to receive quality of life questionnaires and to be randomized according to the TwiCs design, are eligible to be included in the ARCADE trial.

### Eligibility criteria {10}

Patients are eligible for randomization if they meet all the following criteria:

1. Participation in the PACAP cohort with written informed consent for quality of life questionnaires (patient-reported outcome measures; PROMs) and broad consent for future randomization (TwiCs)
2. Histologically proven local recurrence after primary resection. Regional lymph node metastases are also considered local recurrence. In case histological confirmation cannot be obtained (because the first attempt fails or since it is technically impossible), consensus on presence of isolated local recurrence may be obtained in a multidisciplinary meeting (e.g., based on imaging, elevated cancer antigen 19-9 (CA 19-9), and clinical situation)
3. Eastern Cooperative Oncology Group (ECOG) performance score 0–2

Exclusion criteria are as follows:

1. Distant metastases at recurrence diagnosis. Regional lymph nodes are not considered distant metastases.
2. Expected lifespan < 3 months
3. Previous radiotherapy precluding SBRT
4. Highly selective cases eligible for re-resection without induction therapy, according to the expert panel
5. Age < 18 years
6. Pregnancy

### Who will take informed consent? {26a}

According to the TwiCs design, only patients randomized for the intervention group are approached by the radiation oncologist from one of the three participating radiation centers to obtain additional informed consent.

### Additional consent provisions for collection and use of participant data and biological specimens {26b}

Participants included in the ARCADE trial have provided informed consent to collect their data in a standardized fashion in context of PACAP. According to the TwiCs design, only patients randomized for the intervention group are asked to provide informed consent for participation in the ARCADE trial. This trial does not involve collecting biological specimens.

## Interventions

### Explanation for the choice of comparators {6b}

All patients (in both study groups) will receive standard of care. In most cases, this comprises standard chemotherapy, either consisting of 12 cycles of (modified) leucovorin, 5-fluorouracil (5-FU), irinotecan, and oxaliplatin combination therapy (FOLFIRINOX), consisting of a 5-FU bolus 400 mg/m^2^ on day 1 and continued infusion of 2400 mg/m^2^ during 46 h; oxaliplatin 85 mg/m^2^ on day 1; irinotecan 180 mg/m^2^ on day 1 and leucovorin 400 mg/m^2^ on day 1, or 6 cycles of gemcitabine (1000 mg/m^2^) and Nab-Paclitaxel (125 mg/m^2^) on days 1, 8, and 15 of a 4-week cycle. If patients refrain from chemotherapy, out of choice or because of impaired physical condition, patients will receive best supportive care (BSC).

### Intervention description {11a}

Additional SBRT will be administered to patients in the intervention group following an image-guided, hypo-fractionated scheme of 5 fractions of 8 Gray (Gy), prescribed to 95% of the planning target volume. The gross tumor volume is the local recurrent PDAC as defined on either CT or MRI. SBRT is delivered in one of the three participating centers. In the UMC Utrecht and Amsterdam UMC, SBRT is applied using MR guidance. In the Erasmus MC, CT-guided imaging is used and therefore three radiopaque markers (fiducials) are placed in or near the tumor [55]. Treatment is delivered on alternate days 2 or 3 times a week with a maximum overall treatment time of 21 days in one of the participating centers. Treatment preparation and delivery procedures will be determined in accordance with the protocols of the treating center. However, the irradiation dose constraints for all organs at risk will be the same for all institutions.

#### Concurrent treatment

Patients randomized for SBRT can receive the intervention in addition to standard of care, which mostly comprises systemic chemotherapy. There is no preset order in which both treatments are applied. However, they cannot be received simultaneously. SBRT will be given as early as possibly after diagnosing isolated local recurrence. Hence, if systemic chemotherapy treatment has already started, SBRT can be delivered between two cycles of systemic therapy.

### Criteria for discontinuing or modifying allocated interventions {11b}

Patients can leave the study at any time for any reason if they wish to do so without any consequences. The steering committee may withdraw a patient from the study for one or more of the following reasons: (1) incorrect randomization, meaning that the eligibility criteria were not followed correctly, (2) continuing participation could be harmful to the patient, (3) the study is stopped early. An individual patient will not be replaced by another patient after withdrawal, but they will be followed up by a medical doctor until death.

### Strategies to improve adherence to interventions {11c}

To improve adherence to the intervention protocol, patients can freely choose in which of the three participating radiotherapy centers they want to undergo the intervention. After SBRT and corresponding follow-up appointments, patients can go back to their referring hospital to receive further treatment and/or follow-up.

### Relevant concomitant care permitted or prohibited during the trial {11d}

Other experimental treatment is only prohibited during the radiotherapy intervention of patients.

### Provisions for post-trial care {30}

Participants will remain enrolled in the ARCADE trial for a maximum of 18 months or until patient withdrawal. After their participation in the ARCADE trial, participants will be referred to their treating medical oncologist or general practitioner.

### Outcomes {12}

The primary study endpoint is survival after recurrence by intention-to-treat, defined as the time between the date of recurrence diagnosis and the date of death from any cause or date of last follow-up. The date of histological evidence of disease recurrence will be used as the date of recurrence diagnosis. In case histological evidence could not be obtained, the date of the multidisciplinary meeting in which isolated local recurrence was diagnosed will be used.

Secondary endpoints are:

- Patient-reported quality of life, as standardly measured by assessing PROMs as a part of the current PACAP of the Dutch Pancreatic Cancer Group (DPCG) [51].
- Treatment response, assessed by CT imaging according to Response Evaluation Criteria in Solid Tumors v1.1 (RECIST) [56].
- Acute and late toxicity, as assessed using Common Terminology Criteria for Adverse Events (CTCAE) version 5.0 during regular follow-up moments [57]. In the intervention arm, acute toxicity will be monitored by the treating radiation oncologist. Acute toxicity will be defined as toxicity within 90 days from the end of SBRT treatment and will be assessed in weeks 1, 3, 6, and 12. Late toxicity is defined as toxicity occurring > 90 days from SBRT.
- Overall survival, defined as the interval between the date of primary resection and the date of death from any cause.
- Progression-free interval, defined as the interval between the date of disease recurrence and the date that local and/or distant progression of disease occurs.
- Local progression-free interval, defined as the interval between the date of disease recurrence and the date that locoregional progression of disease occurs.
- Distant metastasis-free interval, defined as the interval between the date of disease recurrence and the date that distant progression of disease occurs.
- To assess patients’ acceptability of SBRT

### Participant timeline {13}

Table 1 shows the participant timeline.

**Table 1:** Schedule of enrolment, intervention, and assessments according to the SPIRIT guidelines

Timepoint	Study period				
	Identification	Allocation	Post-allocation		
	*Recurrence diagnosis*		*pre-SBRT*	*SBRT*	*After completing therapy*^*d*^
PACAP registry, TwiCs, and PROMs informed consent	X				
Histological confirmation PDAC recurrence	X				
Informed consent for additional SBRT (investigational arm)		X			
SBRT 5x8 Gy				X	
History and physical examination			X		Every 3 months
Laboratory investigation including tumor markers			X		Every 3 months
MRI scan chest and abdomen			X^b^		
CT scan chest and abdomen			X		At 3, 6, and every subsequent 6 months
Fiducial marker-placing			X^c^		
QoL questionnaires^a^	X				At 3, 6, 9, 12, and 18 months after recurrence diagnosis

^a^The following questionnaires are used: general questions, questions on exocrine pancreas insufficiency (EPI), non-disease specific health-related quality-of-life (HRQL) (EQ-5D-5L), cancer-specific HRQL (EORTC QLQ-C30), tumor-specific HRQL (EORTC QLQ-PAN26), neuropathy (EORTC QLQ-CIPN20), happiness, hospital anxiety and depression scale (HADS), worry of progression of cancer scale (WOPS; modified from ways of coping scale (WOCS)

^b^Only for patients treated at the UMC Utrecht or Amsterdam UMC

^c^Only for patients treated at the Erasmus MC

^d^For patients receiving chemotherapy, this will be after their chemotherapy. During chemotherapy they will be treated according to standard of care. For patients receiving best supportive care, this will be after radiotherapy

### Sample size {14}

The pooled median survival in patients with isolated local PDAC recurrence is 9 months from the time of recurrence; the pooled median survival of patients additionally treated with SBRT is 16 months [10, 19, 22, 47, 48, 58, 59].

As we expect 80% of patients to accept the experimental intervention offered in the intervention group, an estimated refusal rate of 20% needs to be taken into account. This dilutes the overall survival rate to 14.6 months (80% × 16 months + 20% × 9 months) for all patients in the intervention group. The clinically relevant survival difference of 14.6 months vs. 9 months for respectively the intervention and control group corresponds to a relative hazard (RH) of survival of 1.62, which was used to calculate the sample size of the study.

To detect a 62% improvement (RH of survival of 1.62) in overall survival for patients in the intervention group, as compared with the control group, with a statistical power of 80% and a 0.05 two-sided significance level, a sample size of 174 patients is required. This calculation was based on the assumption of an exponential model, a median overall survival of 9 months in the control group, a follow-up duration of 18 months, a censoring rate of 1%, and a baseline event rate of 7.7%. Following this calculation, we plan to include 174 patients in total: 87 patients in the control group and 87 patients in the intervention group [60].

### Recruitment {15}

Nationwide collaboration within the DPCG will enhance patient enrolment. In the Netherlands, treatment of patients with isolated PDAC recurrence takes place in centers affiliated with the DPCG. Our goal is to include all 15 DPCG-affiliated centers in this study.

In 2019, 360 patients underwent macroscopically radical (R0-R1) resection of a PDAC in the Netherlands. All pancreatic resections in the Netherlands are performed in centers affiliated with the DPCG. An earlier study showed that 21% of these patients develop isolated local recurrence [61]. However, previous trials conducting a trial-specific, standardized surveillance strategy showed that with standardized surveillance, isolated local recurrence can be found in 26% of patients (Table 2). As the RADAR-PANC trial on the additional value of a three-monthly standardized surveillance with imaging and tumor marker testing will be conducted simultaneously within the Netherlands (NCT04875325), isolated local recurrence is expected in about 25% of patients (*n* = 90). Based on the current successful enrolment progress, we anticipate that 90% (*n* = 81) of all patients will be registered yearly in the PACAP cohort and that 83% (*n* = 67) of these patients will provide informed consent for the TwiCs design, based on the current PACAP participation rate. Fifty percent of these patients (*n* = 33) will be randomized to the intervention arm of the trial. The expected time needed for inclusion of a total of 174 patients (87 patients in each arm) will be 4.5 years, including a start-up period of 1.5 years in which the trail is initiated in all DPCG centers. Besides, during this start-up phase of the trial, isolated local recurrence rates will be lower due to the fact that the standardized follow-up protocol initiated by the RADAR-PANC trial is not yet rolled out in all centers. The final analysis will be performed 18 months after the last patient is enrolled.

**Table 2:** Incidence of isolated local recurrence after resection for pancreatic cancer in selected randomized controlled trials on adjuvant therapy

Reference and name of the study	***N***	Incidence of ILR
Neoptolemos et al. (2004); ESPAC-1 [4]	289	35%
Smeenk et al. (2007) EORTC 40891 (long-term results) [21]	218	21%
Regine et al. (2008); RTOG 97-04 [15]	451	26%
Ueno et al. (2009); JSAP-02 [16]	118	28%
van Laethem et al. (2010); EORTC-40013-22012/FFCD-9203/GERCOR [17]	90	18%
Uesaka et al. (2016); JASPAC 01 [18]	377	23%
**Pooled incidence**		**26%**
**National recurrence database** [61]		**21%**

## Assignment of interventions: allocation

### Sequence generation {16a}

Participants are randomly allocated by a computer-generated program, following 2-4-6 block randomization. Randomization will be stratified by institute and surveillance strategy. This can be symptomatic, according to current clinical practice, or standardized, for example when a patient is participating in the RADAR-PANC trial (TwiCs to investigate the impact of a standardized surveillance strategy using imaging and serum tumor marker testing on survival and quality of life in patients who underwent resection of PDAC; NCT04875325).

### Concealment mechanism {16b}

According to the TwiCs design, only participants randomized for the intervention will be informed about their randomization. Directly after randomization, these patients will be contacted by the radiation oncologist to inform them that they have been randomized for the intervention. Patients randomized for the control group will not be informed. Therefore, there is no need to conceal participants’ allocation.

### Implementation {16c}

Participants are randomized as soon as they meet all of the inclusion criteria. The central study coordinator performs the randomization and allocates participants to the intervention. The pros and cons of SBRT will be explained and additional informed consent will be asked. Patients not giving informed consent for SBRT will be followed and analyzed according to the intervention arm (ITT). When a participant is randomized for the intervention arm, the radiation oncologist from one of the three radiation centers (based on patient preference) will be informed. The radiation oncologist will contact the patient to schedule an appointment to inform them about the intervention.

## Assignment of interventions: blinding

### Who will be blinded {17a}

Blinding is not applicable to studies that are designed according to the TwiCs design. However, as inherent to the design, participants randomized to the control group will not be informed explicitly.

### Procedure for unblinding if needed {17b}

Not applicable since blinding will not be performed.

## Data collection and management

### Plans for assessment and collection of outcomes {18a}

Baseline characteristics of all trial participants are standardly collected as part of the PACAP cohort of the DPCG. Also, quality of life is assessed at standard time points in all PACAP participants by the PROMs. Additional data is collected from the patients’ electronic files. Local clinicians in the participating centers are responsible for data collection. They can, however, transfer this responsibility to the study team. The study team will appoint appropriate personnel for data collection.

### Plans to promote participant retention and complete follow-up {18b}

The treating radiation oncologist will schedule follow-up appointments according to protocol to keep participants randomized for the intervention in the trial and complete their follow-up. Additionally, the central study coordinator will closely follow all trial participants during their follow-up.

### Data management {19}

Data management will be carried out in accordance with the UMC Utrecht data management policy in accordance with the predefined data management plan. Data will be collected using a predefined electronic case report form in Castor EDC, containing only coded data.

### Confidentiality {27}

The handling of personal data will comply with the Regulation (EU) 2016/679 of the European Parliament and of the Council of 27 April 2016 on the protection of natural persons regarding the processing of personal data and on the free movement of such data (General Data Protection Regulation). A subject identification code list will be used to link the data to the subject. These codes will not be based on the patient initials and birth date. The local investigator will safeguard the key to this code.

### Plans for collection, laboratory evaluation, and storage of biological specimens for genetic or molecular analysis in this trial/future use {33}

As previously stated in 26b, there will be no biological specimens collected.

## Statistical methods

### Statistical methods for primary and secondary outcomes {20a}

Baseline data will be analyzed and reported using standard descriptive statistics. Randomization success will be evaluated by comparing baseline data of the intervention group to the control group. Analyses will be performed according to the intention-to-treat principle. The primary endpoint is survival after recurrence, defined as the time between the date of recurrence diagnosis and the date of either death from any cause or last follow-up. Survival after recurrence, as well as the secondary endpoints overall survival, progression-free interval, local progression-free interval, and distant metastases-free interval, will be reported as median with 95% confidence interval (CI) and will be calculated using the Kaplan-Meier survival curve method. Log-rank test will be used to compare groups. In addition, a sensitivity analysis will be conducted on survival after recurrence, whereby this will be defined as the time between the date of randomization and the date of death from any cause or last follow-up. Univariate cox-proportional hazard analysis will be performed to determine the crude effect of SBRT on survival after recurrence. Multivariable analysis will be performed to determine adjusted effect estimates. The adjusted analysis will be corrected for several baseline confounding factors, such as age, sex, preoperative CA 19-9 level, tumor size, number of positive lymph nodes, tumor differentiation, resection margin status, adjuvant chemotherapy, and treatment for disease recurrence. Results will be presented as HRs with corresponding 95% CIs. A two-tailed probability value (*P*-value) of < 0.05 is considered statistically significant. Treatment response, acute and late toxicity, and reasons for non-eligibility or exclusion will be reported using descriptive statistics. Chi-square or Fisher’s exact test are used to compare categorical variables as appropriate. Parametric continuous variables are presented as mean with standard deviation (SD) and are compared using Student’s *t* test. Non-parametric continuous variables are presented as median with interquartile range (IQR) and are compared using the Mann-Whitney *U* test. Acute and late toxicity will also be evaluated by a mixed model, to account for both within-person and across-person variability and to take repeated toxicity measurements into account. Baseline quality of life will be compared to all other time points during follow-up. A change of 10% of the scale width will be considered a clinically relevant change of quality of life [62]. The data will be presented as stable, worsened (≥10% decrease in quality of life), or improved (≥10% increase in quality of life). These time points will be compared using a chi-square test with a *P*-value of ≤0.05. We will also evaluate the pattern of quality of life as continuous outcome over time during follow-up with repeated measurement analysis using the mixed-models approach.

The latest version of R Studio will be used for statistical analysis.

### Interim analyses {21b}

Interim analysis on efficacy will be conducted when 50% of the required patient number (87 patients) is included, and of which, the patients randomized for the intervention have received SBRT. Results will be shared with the Medical Research Ethics Committee (MREC), which can decide to prematurely end the study. Stopping guidelines are a twofold increase in the primary endpoint (survival after recurrence) of the intervention group compared to the control group, or more than 20% refusal of the intervention. A *P*-value of < 0.01 will be considered statistically significant.

### Methods for additional analyses (e.g., subgroup analyses) {20b}

When sufficient number of patients are available within the following subgroups, comparisons will be made between patients who received SBRT + chemotherapy, SBRT alone, chemotherapy alone, and BSC alone, and the impact on survival outcomes and quality of life within these specific subgroups will be assessed. Assuming a 0.50 minimum relevant effect size, statistical power of 80%, and a 0.05 two-sided significance level, a subgroup should contain at least 33 patients to detect the effect size.

### Methods in analysis to handle protocol non-adherence and any statistical methods to handle missing data {20c}

Since some patients will deny the intervention after being randomized (according to the TwiCs design), a refusal rate was taken into account when calculating the sample size. Analyses will be performed according to the intention-to-treat principle, which means that participants who are randomized for the intervention group but deny this intervention will therefore be analyzed as if they did receive the intervention. Missing baseline data will be imputed using multiple imputation techniques. Both complete case analysis and analysis after multiple imputation will be performed to check for inconsistencies.

### Plans to give access to the full protocol, participant-level data, and statistical code {31c}

The study protocol, derived data, and statistical analysis code will be made available upon request.

## Oversight and monitoring

### Composition of the coordinating center and trial steering committee {5d}

At the coordinating center, a Doctor of Philosophy (PhD) student is responsible for running the trial on a day to day basis, supervised by two principal investigators and a postdoctoral researcher. At least once a week, trial progress is being evaluated and during the week supervisors can be consulted for additional deliberation. Also, (potential) trial participants are identified and discussed during weekly multidisciplinary team meetings.

At all three participating radiation centers, the local principal investigators (radiation oncologists) are responsible for the on-site logistics. The Trial Steering Committee contains members of the DPCG, who can easily be updated on the progress of the trial during the four yearly meetings but can be additionally updated upon request. Data management by the trial team is supported by data managers from the coordinating.

### Composition of the data monitoring committee, its role and reporting structure {21a}

The quality of the study is monitored by Julius Clinical, an independent science contract research organization, which will control the safety of trial subjects. The assigned monitor will check inclusion and dropout rates, completeness of study documents and informed consents, information on serious adverse event (SAE) procedures, in- and exclusion criteria, study procedures, and personnel certification and training, Initiation visits will be scheduled before commencing including in each participating center. Following monitoring visits will be scheduled after the first five inclusions, and consequently at least two times a year per center (depending on patient enrolment). In the end, a close-out visit will be scheduled in each participating center.

### Adverse event reporting and harms {22}

All grade 3 or higher (S)AEs (either expected or unexpected) reported spontaneously by the subject or observed by the site investigator, or his staff will be recorded up to 3 months after SBRT in the corresponding section of the electronic case report form. SAEs will be reported by the local principal investigator or his staff within 24 h of becoming aware of the SAE to the UMC Utrecht principal investigator in encrypted form by means of the SAE form. The sponsor will then report the SAEs through the web portal *ToetsingOnline* to the accredited MREC that approved the protocol, within 7 days of first knowledge for SAEs that result in death or are life threatening followed by a period of maximum of 8 days to complete the initial preliminary report. All other SAEs will be reported within a period of maximum 15 days after the sponsor has first knowledge of the SAE. After the first 3 months after treatment, only the treatment induced grade 3 or higher (S)AEs will be recorded up to the end of the study. The principal investigator or an authorized delegate will decide whether an (S)AE is related to the SBRT. As PDAC patients have a very poor prognosis, we expect that many patients suffer from follow-up radiotherapy unrelated SAEs within the 2-year study period. These SAEs will be recorded, although not reported.

### Frequency and plans for auditing trial conduct {23}

At any given point during the study, the trial can be selected for audit. There is no predefined schedule for audits and inspections.

### Plans for communicating important protocol amendments to relevant parties (e.g., trial participants, ethical committees) {25}

During the trial, the sponsor might want to make changes to the protocol or other trial documents which require a new favorable opinion by the competent authority and MREC. If necessary, trial participants will be informed about these amendments and updated Informed Consent might be obtained. All amendments will be communicated to the participating trial centers.

### Dissemination plans {31a}

Trial results will be fully disclosed by means of publication in peer-reviewed journal and by presentations at national and international scientific meetings. Both positive and negative findings will be disclosed.

## Discussion

The ARCADE trial investigates whether SBRT in addition to standard of care improves survival after recurrence and quality of life compared to standard of care alone, in patients with isolated local recurrence after resection of PDAC in the Netherlands.

During the design of the study, there were several points of discussion. First, there has been some debate about the exact timing of randomization. All patients with isolated local recurrence in the Netherlands will be offered to receive chemotherapy as recurrence treatment. The initial idea was that patients with isolated local recurrence would first have to undergo several courses of systematic chemotherapy. After these courses, tumor evaluation would take place, and in case the tumor was stable and distant metastases did not develop, patients would be randomized. However, this entails the risk that patients who were initially eligible for SBRT will develop distant metastases during chemotherapy and therefore can no longer undergo SBRT for isolated local recurrence. Additionally, some patients are unwilling or unable to undergo chemotherapy, while they do wish to receive radiotherapy for local tumor control. Consequently, it has been decided that patients who are randomized for the intervention group will receive SBRT in addition to standard of care, regardless of the facts whether this entails systematic chemotherapy or BSC. On the basis of randomization, it can be expected that the ratio of patients who do or do not undergo chemotherapy is the same in both groups.

Finally, thoughts were exchanged about the TwiCs design. In a conventional randomized controlled trial, patients want to avoid the possibility of being randomized for the control group and therefore renounce participation. More so, if they do decide to participate, they may be disappointed when randomizing for the control group, causing them to drop out or crossover to the intervention group. In case of the TwiCs design, however, patients randomized for the control arm will not be notified explicitly, which limits selection and crossover bias and prevents potential distress in patients that are being randomized for the control group. Furthermore, it enables efficient use of yet existing data being routinely collected by means of the cohort. As a result, new interventions can be made available to eligible patients more quickly [54]. Since patients can be identified from a yet existing cohort, patient recruitment is more effective. Finally, patients’ acceptability towards the intervention can be measured. The fact that participants randomized for the control arm are not specifically informed of their participation in a specific trial raised some concerns amongst physicians. However, a recent study has shown that patients themselves did not experience this as a problem [63].

## Trial status

Protocol version 2.0, February 4, 2021. The first participant was randomized on July 5, 2021. Recruitment is anticipated to be completed at the end of 2025 (see NCT04881487 on clinicaltrials.gov for the active recruitment status of the trial).

## Data Availability

The study protocol, derived data, and statistical analysis code will be made available upon request.

## Abbreviations

5-FU: 5-Fluorouracil
(S)AE(s): (Serious) adverse event(s)
BSC: Best supportive care
CA 19-9: Cancer antigen 19-9
CI: Confidence interval
CT: Computed tomography
CTCAE: Common Terminology Criteria for Adverse Events
DPCG: Dutch Pancreatic Cancer Group
ECOG: Eastern Cooperative Oncology Group
EDC: Electronic data capture
EPI: Exocrine pancreas insufficiency
FOLFIRINOX: Chemotherapy combination therapy consisting of leucovorin, 5-fluorouracil, irinotecan and oxaliplatin
Gy: Gray; the absorption of one joule of radiation energy per kilogram of matter
HADS: Hospital Anxiety and Depression Scale
HRQL: Health-related quality of life
IQR: Interquartile range
MC: Medical center
MREC: Medical Research Ethics Committee
MR(I): Magnetic resonance (imaging)
PACAP: The Dutch Pancreatic Cancer Project
PDAC: Pancreatic ductal adenocarcinoma
PhD: Doctor of Philosophy
PROMs: Patient-reported outcome measures
*P*-value: Probability value
RECIST: Response evaluation criteria in solid tumors
RH: Relative hazard
SBRT: Stereotactic body radiation therapy
SD: Standard deviation
TwiCs: Trials within Cohorts
UMC: University Medical Center
WOCS: Ways of Coping Scale
WOPS: Worry of Progression of Cancer Scale
