# Correction: A nationwide randomized controlled trial on additional treatment for isolated local pancreatic cancer recurrence using stereotactic body radiation therapy (ARCADE)

**DOI:** 10.1186/s13063-022-07036-8

**Published:** 2023-01-24

**Authors:** I. W. J. M. van Goor, L. A. Daamen, M. G. Besselink, A. M. E. Bruynzeel, O. R. Busch, G. A. Cirkel, B. Groot Koerkamp, N. Haj Mohammed, H. D. Heerkens, H. W. M. van Laarhoven, G. J. Meijer, J. Nuyttens, H. C. van Santvoort, G. van Tienhoven, H. M. Verkooijen, J. W. Wilmink, I. Q. Molenaar, M. P. W. Intven, for the Dutch Pancreatic Cancer Group

**Affiliations:** 1 Department of Surgery, Regional Academic Cancer Center Utrecht, Utrecht, the Netherlands; 2 Department of Radiation Oncology, Regional Academic Cancer Center Utrecht, Utrecht, the Netherlands; 3 Division of Imaging and Oncology, University Medical Center Utrecht, Utrecht, the Netherlands; 4 Department of Surgery, Amsterdam University Medical Center, University of Amsterdam, Amsterdam, the Netherlands; 5 Cancer Center Amsterdam, Amsterdam, the Netherlands; 6 Department of Radiation Oncology, Amsterdam University Medical Center, location Vrije Universiteit, Amsterdam, the Netherlands; 7 Department of Medical Oncology, Regional Academic Cancer Center Utrecht, Utrecht, the Netherlands; 8 Department of Surgery, Erasmus Medical Center, Rotterdam, the Netherlands; 9 Department of Radiation Oncology, Radboud University Medical Center, Nijmegen, the Netherlands; 10 Department of Medical Oncology, Amsterdam University Medical Center, location University of Amsterdam, Amsterdam, the Netherlands; 11 Department of Radiation Oncology, Erasmus Medical Center, Rotterdam, the Netherlands; 12 Department of Radiation Oncology, Amsterdam University Medical Center, University of Amsterdam, Amsterdam, the Netherlands

**Correction: Trials 23, 913 (2022)**

**https://doi.org/10.1186/s13063-022-06829-1**

The original publication of this article [1] contained 2 redundant affiliations in the author information and in the “administrative information table”. These redundant affiliations (previously numbered nr. 2 and 10) have been deleted. The publisher apologizes to the author for the inconvenience caused.
